# Infants Investigated by the Child Welfare System: Exploring a Distinct Profile of Risks, Service Needs, and Referrals for Support in Ontario

**DOI:** 10.3390/brainsci7080101

**Published:** 2017-08-12

**Authors:** Joanne Filippelli, Barbara Fallon, Esme Fuller-Thomson, Nico Trocmé

**Affiliations:** 1 Factor-Inwentash Faculty of Social Work, University of Toronto, 246 Bloor Street West, Toronto, ON M5S 1V4, Canada; barbara.fallon@utoronto.ca (B.F.); esme.fullerthomson@utoronto.ca (E.F.-T.); 2 Centre for Research on Children and Families, McGill University, 3506 University Street, Montreal, QC H3A 2A7, Canada; nico.trocme@mcgill.ca

**Keywords:** child welfare, infants, referral to services, child maltreatment

## Abstract

The science of early childhood development underscores that maltreatment and other adversities experienced during infancy heightens the risk for poor developmental and socio-emotional outcomes. Referrals to supportive services by the child welfare system are particularly critical during infancy given the rapidity of brain development and infants’ sensitivity to their environment. The main objectives of the current study are to: (1) examine age-specific differences in clinical and case characteristics; (2) determine the factors associated with the service referral decision involving infants; and (3) explore the types of services families have been referred to at the conclusion of a maltreatment-related investigation. Using data from the Ontario Incidence Study of Reported Child Abuse and Neglect for 2013, descriptive analyses were conducted, as was a logistic regression to identify factors associated with the decision to refer families of infants to supportive services. Overall, the findings reveal that the profile of infants and their families differs distinctly from those of older children with respect to risks, service needs, and service referrals, although this is rarely reflected in child welfare practice and policy. Investigations involving infants were most likely to have a referral made to supportive services, least likely to have an infant functioning concern identified; most likely to have a primary caregiver risk factor identified; and, the greatest likelihood of experiencing economic hardship. Multiple risks, identified for the primary caregiver of the infant are correlated to referral decisions for infants. However, the needs of the infant are likely under-identified and require cross-sectorial collaboration.

## 1. Introduction

Children and families investigated by the child welfare system often have complex needs that may require various types of supportive services that span across numerous sectors [1,2,3,4]. The decision to refer children and families to supportive services can signal the child welfare systems’ recognition of child and/or family need [5]. A child welfare worker’s decision to refer to services is predicated upon the assessment of need. Identifying child and family needs and aligning services with those needs may help to prevent deterioration in family functioning, decrease the risk of maltreatment and increase the likelihood of family reunification [4,6,7]. Service referrals are an important step in promoting both the safety and well-being of children and can be particularly consequential for infants and young children. Early identification, referral, and intervention may buffer or prevent the developmental consequences of maltreatment and other adversities. Without appropriate intervention, developmental difficulties that emerge in early childhood can become more challenging to address and ameliorate over time [8]. Early identification and intervention are critical as the brain is most receptive to the environment in the first years of life [9]. Yet, there is minimal literature focusing on service referrals within the context of child welfare service provision [10]. There is little understanding of the types of services that children and families are referred to, including referrals to concrete services that may assist families in meeting their basic needs (e.g., food, housing, utilities), educational services (e.g., parent support groups), and clinical services (e.g., mental health counseling) [1,10]. There is no study that has explored the factors associated with the decision to provide service referrals to families of infants; nor, is there a study that has explored the types of services that families of infants and older children are referred to within the context of maltreatment-related investigations in Canada. 

### 1.1. Developmental Issues for Infants

A high rate of developmental concerns has been found in infants and toddlers regardless of whether allegations were substantiated or unsubstantiated [11,12]. Children who remain in the home have shown similar high rates of mental health issues as children placed into out-of-home care [13,14]. For infants and young children involved with the child welfare system, there is a significant gap between the identification of need for mental services and service receipt [14]. Child welfare-involved infants are unlikely to have their developmental and mental health needs met prior to school entry [15]. Infants reported to and investigated by the Ontario child welfare system have been noted to be the least likely group of children to be identified by child welfare workers as having a functioning concern [16]. The lack of identification of developmental issues by the child welfare system can translate into low referral rates to, and underuse of, early intervention services [17,18]. There have been concerns about the adequacy of the child welfare system’s response to the unique needs of infants and young children throughout the extant literature [11,18,19,20,21,22,23].

### 1.2. Caregiver Concerns

Using the 2008 cycle of the Canadian Incidence Study of Reported Child Abuse and Neglect (CIS-2008), Jud, Fallon, and Trocmé found that caregiver risk factors (e.g., few social supports), younger caregiver age, and socio-economic hardship were significantly associated with the likelihood of receiving ongoing child welfare services or a referral to services post-investigation [24]. Palusci explored post-investigation services and recurrence after confirmed psychological maltreatment for children and found that poverty, drug, or alcohol issues and other forms of violence increased the likelihood of a service referral [25]. Less than one quarter of families were referred to services following confirmed psychological maltreatment.

Caregivers of children involved with the child welfare system have also been noted to experience unmet service needs [26,27]. In Ontario, being a victim of intimate partner violence (IPV), having few social support (i.e., social isolation or lack of social supports), and mental health issues have consistently emerged as the most frequently identified caregiver risk factors in maltreatment-related investigations involving infants [16,28]. Access to social supports from the community can help to buffer stress and reduce social isolation, a risk factor for both infant maltreatment and poor infant psychosocial functioning [29,30]. The importance of social supports in child welfare decision-making is highlighted by research indicating that a caregiver with few social supports is an influential predictor for transferring a case to ongoing child welfare services in two studies exploring maltreatment-related investigations involving infants and their families in Ontario [16,28]. A caregiver’s social support network is particularly consequential for infants as it can impact the quality of infant-caregiver relationship [30]. 

### 1.3. Factors Associated with Service Referrals and Dispositions

Child welfare workers have been described as service brokers and gateway providers to services for children and youth (e.g., [15,31,32]). A referral made by a child welfare worker for support services is a critical step to match services to needs [33]. The importance of understanding and addressing the needs of infants and young children who come into contact with the child welfare system is underscored by the accumulating evidence of their developmental vulnerabilities, unmet needs, and the underutilization of services [14,15,20]. Research suggests that initial involvement with the child welfare system may increase access to mental health services [15].

Despite the consequential nature of the service referral decision to the provision of services, child welfare decision-making research has primarily focused on the provision of ongoing child welfare services [16,24,28,34] and the decision to place children out-of-home [24,35,36,37]. The applicability and generalizability of the available extant service referral research to the Ontario child welfare context is limited by the lack of uniformity in several aspects of studies, including methodology, sample, variable type, definition of referral to services, the types of services referred to, the country of origin, and the broader policy and practice contexts. For example, Villigrana examined the factors that influence a referral to mental health services for child welfare-involved children by both court social workers and child welfare social workers in California by using case abstraction of closed court cases [38]. There were no significant predictors found for the decision to refer children to mental health services by child welfare social workers; however, significant predictors for mental health service referrals for court social workers have included the child remaining in their home and multiple types of abuse. Factors associated with service referrals have been found to differ by ethnicity [5]. For instance, a substantiation decision was more likely to result in a service referral for white families than for black families [5]. When compared to African American families, Hispanic families have been found to be more likely to be referred to psychosocial services than services that relate to basic needs, such as housing [10]. Amongst children with a developmental disability, a report of emotional maltreatment and out-of-home placement were two factors that increased the likelihood of a service referral for a formal assessment [39]. The likelihood of a referral to developmental services was more likely for cases in which the child welfare system had determined a need for services and where children experienced sexual abuse [40].There is a dearth of research that explores service referrals for infants and young children.

### 1.4. International Comparisons and Context

The majority of research exploring service referrals and/or utilization for child welfare-involved families originates from the United States, which has disparate policy, legislative, and practice contexts than Ontario, Canada. For instance, there are differences between the U.S. and Canada in how the knowledge of early childhood development has informed child welfare legislation and policies. In the U.S., amendments were made in 2003 to the Child Abuse and Prevention Act (CAPTA) in recognition of the importance of early intervention for young child welfare-involved children [11,40]. Funded under Part C of the Individuals with Disabilities Education Improvement Act (IDEA), states were required to develop referral procedures to early intervention services for children aged 0 to 3 years who were involved in a substantiated case of child maltreatment [11,40]. In 2011, the Child and Family Services Improvement and Innovations Act was introduced and this legislation required state reporting of activities involving young children in the child welfare system [41]. Moreover, in contrast to the U.S., child welfare in Canada is not governed by federal legislation, but legislation that is specific to each province and territory [42,43]. Ontario’s child welfare legislative mandate gives central and equal consideration to the notions of both protection and well-being [44]; however, there are no policies that address early intervention for infants and young children who come into contact with the child welfare system, nor is there mandated referral legislation. Although there is an existing policy orientation in support of differential response models, wide-scale programs have yet to be implemented in Ontario [16,45]. In contrast, several states in the U.S. have undergone Differential Response reforms, which emphasize family assessment, parental involvement, and needs-driven service [46]. In comparison to families that undergo investigations, families that are offered services through an assessment approach have been found to be provided with a greater array of services [46]. Child welfare service models in Canada are not yet aligned with emerging investigative trends that suggest greater focus on the long-term impact of family dysfunction than immediate safety concerns [44]. 

Findings from the research on Canadian incidence studies at both the provincial and national levels suggest that infants are a unique subgroup of children involved with the child welfare system [16,28,34,37]. The age of children influences the risk of maltreatment and how the child welfare system subsequently responds to it [23,25,47]. Investigations involving infants are most likely to result in intensive service responses, including greater likelihood of receiving ongoing child welfare services and out-of-home placements [37]. Key drivers of service provision decisions differ by age. Caregiver risk factors have been found to drive the decision to provide ongoing child welfare services [16,28,34] and out-of-home placements for infants [48]. In contrast, child functioning concerns are key contributors for both decisions involving older children and adolescents [36,37]. These findings underscore the importance of exploring the unique mix of child, family, and broader environmental risks, protective factors, and needs that may emerge around specific ages and developmental stages [47]. The composition of services required to support families with infants should differ from those required to support families with older children [47]. Palusci found that infants and young children (0–5 years of age) involved with the child welfare system experienced different risk factors and services than those of older children [49]. There is a dearth of research exploring types of services offered to families who have had involvement with the child welfare system [10].

### 1.5. Research Questions

The state of the literature suggests that the child welfare decision to refer families to services warrants more focused attention, particularly given the salience of this decision to infants and their families within a Canadian child welfare context. There is no study that has specifically examined the service referral decision for infants and families who have been investigated by the child welfare system; nor, has there been an age-specific exploration of the patterns and types of services families have been referred to in a Canadian context. An understanding of the clinical profile infants and their families, their needs, and the child welfare system’s response is critical to developing targeted, appropriate, and effective interventions. As a result of the significant gaps in the knowledge base, this exploratory study uses the Ontario Incidence Study of Reported Child Abuse and Neglect-2013 (OIS-2013) [50] to answer the following questions:

1. What are the characteristics (child, caregiver, household, case, and short-term service outcomes) of maltreatment-related investigations of children that are infants (less than 1) and do these characteristics differ when compared to other age groups, including, preschool aged (1–3), early school-aged (4–7), pre-adolescent (8–11), and adolescent (12–15) children investigated by the child welfare system across Ontario?
2. Which characteristics are associated with the decision to refer infants and families for supportive services following maltreatment-related investigations?
3. What are the types of supportive services that families of infants and older children are referred to?

The OIS is currently the only source of provincially aggregated child welfare data in Ontario. The OIS is also the only source of data that includes whether a child welfare worker made a referral for supportive services during the investigative period. The central objective of this study is to better understand the unique characteristics of infants and their families by exploring differences between infants and other age groups, and to build on the evolving research on factors related to service provision with this particularly vulnerable subpopulation of children. Understanding factors associated with child welfare service referral decisions for infants, and age-specific trends for the broader population of children, is important to strategically addressing and targeting child and family needs. This research provides a snapshot of the risk factors, child and family needs, and the supportive services that families are referred to at the conclusion of an initial child welfare investigation. Given the dearth of research on infants and the decision to provide service referrals within a Canadian child welfare context, this exploratory analysis is warranted and an important step for future research. 

## 2. Materials and Methods

### 2.1. Sample

A secondary analysis of the 2013 cycle of the Ontario Incidence Study of Reported Child Abuse and Neglect (OIS) was conducted. The OIS-2013 is the fifth provincial study to examine provincial estimates of the incidence of reported child maltreatment and characteristics of the children and families investigated by the child protection system in Ontario. The OIS is a serial survey conducted with child welfare workers regarding their investigations of child maltreatment. The study is cross-sectional. The OIS utilizes a three-stage sampling design to select a representative sample of 17 child welfare agencies from a provincial list of 46 child welfare agencies [51]. Cases opened between 1 October 2013 and 31 December 2013 of the study cycle were eligible for inclusion in the study. Children not reported to child welfare services, screened out reports, and new allegations on open cases at the time of selection were not included in the OIS-2013. The three-month study period is considered optimal for participation and compliance with study procedures [51]. 

Maltreatment-related investigations included in the OIS-2013 are comprised of two types: (1) where there is no specific concern about past maltreatment but future risk of maltreatment is being assessed (risk-only); and (2) where maltreatment may have occurred. Maltreatment-related investigations, regardless of their substantiation status were included in this analysis. Children over 15 years of age, siblings not investigated, and children who were investigated for non-maltreatment concerns were excluded from the sample. 

Child welfare cases in Ontario are counted as families. There were 3118 cases opened at the family-level during the 3-month period. The final stage of the sampling consisted of identifying investigated children as a result of maltreatment concerns. There were a total of 5265 children investigated as a result of the identification of maltreatment concerns. Of those 5265 children investigated, 345 were infants. The University of Toronto provided ethics approval (protocol number 28580).

### 2.2. Study Weights

Provincial estimates were derived by applying full weights, which includes both annualization and regionalization weights. These procedures yielded a final weighted sample of 125,281 children investigated because of maltreatment-related concerns. This study focused specifically on maltreatment-related investigations involving infants (under the age of 1 year) and explored factors associated with the decision to provide a referral for supportive services at the conclusion of the investigation. The final provincial estimate was 7915 investigations involving infants. For a detailed description of the OIS-2013 methodology and weighting procedures, please see Fallon and colleagues [51].

### 2.3. Data Collection Instrument

Data for the OIS-2013 is collected directly from investigating child welfare workers using a three-page standardized data collection instrument, the Maltreatment Assessment Form. This form is completed at the conclusion of the initial investigation. The OIS-2013 had an item completion rate of over 99% for all items. The instrument collected clinical information that child welfare workers routinely gather as part of their initial investigation, such as: caregiver, infant, case characteristics, and short-term service dispositions, including whether a referral for supportive services had been made, and the types of services referred to. Child welfare workers were asked to indicate whether any referrals for services had been made for any family member at the end of the investigation. If so, workers were asked to indicate all referrals that applied. These referrals include internal referrals to a special program provided by the child welfare organization or to other agencies or services external to the child welfare organization. 

### 2.4. Variable Selection

The decision to refer to services is a dichotomous variable. The variable definitions and codes used in this analysis are provided in Table 1. Clinical variables were chosen on their availability in the dataset and on the literature addressing factors related to the occurrence of child maltreatment and the child welfare system’s response to infants. 

### 2.5. Analyitic Approach

Descriptive and bivariate analyses were conducted in order to explore and compare the profile of investigations involving infants (under the age of 1) to children in older age groups, including preschool (1–3), and early school-age (4–7), pre-adolescent (8–11), and adolescent (12–15) children in Ontario in 2013. Provincial incidence estimates were calculated by dividing the weighted estimates by the child population based upon 2011 Census data from Statistics Canada. Chi-square tests of significance were conducted using the normalized sample weight, which adjusts for the inflation of the chi-square statistic by the size of the estimate by weighing the estimate down to the original sample size. As Table 2 shows, infants were compared to four other age groups and the p-value was subsequently adjusted as a result (0.05/4 comparisons = 0.013). Thus, all significance tests for chi-square analyses conducted and shown in Table 2 were evaluated using the adjusted *p*-value (*p* < 0.013), resulting from the Bonferroni correction given the four multiple comparisons. 

Multivariate analysis was conducted in order to explore which variables were significant with the decision to provide a service referral at the conclusion of maltreatment-related investigations involving infants. Prior to the logistic regression, bivariate chi-square analyses were conducted to explore associations between clinical and case characteristics and service referrals. Only variables that were significant at the bivariate level (*p* < 0.05) were included in the logistic regression model. Logistic regression was deemed an appropriate analysis strategy as the outcome variable is dichotomous and it can estimate the relationship between predictor variables with the likelihood or probability of an event occurring [52]. The cutoff point for the decision to refer to services was 0.57, which reflects the proportion of investigations referred for services for the infant population. This analysis did not include missing data in the bivariate or multivariate analysis. Unweighted data were used in the multivariate model to ensure unbiased results due to the inflation of significance due to a large sample size. All analyses were conducted using SPSS, version 23 (SPSS Inc., Chicago, IL, USA).

## 3. Results

**Figure 1:**
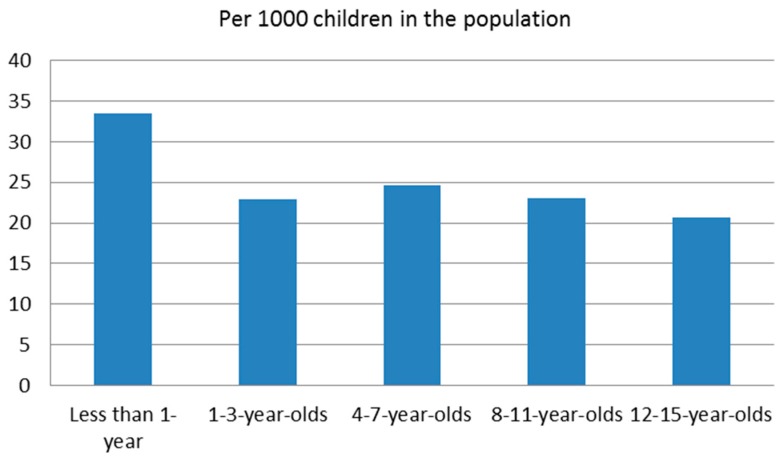
Incidence of a service referral for maltreatment-related investigations by child age.

Numerous distinctions emerged in child, primary caregiver, household, maltreatment, case characteristics, and service outcomes between infants and older children investigated in Ontario in 2013 (Figure 1, Table 2). In comparison to all other age groups, maltreatment-related investigations involving infants had the highest incidence of a service referral at 33.50 per 1000; followed by early school-aged children at 24.69 per 1000; preadolescents at 23.07 per 1000 investigations; pre-schoolers at 22.93 per 1000; and adolescents at 20.66 per 1000 (Figure 1). Incidence rates suggest that investigations involving adolescents were the least likely to result in the decision to provide a service referral for any family member, with a rate of 20.66 per 1000. 

### 3.1. Child Characteristics

**Figure 2:**
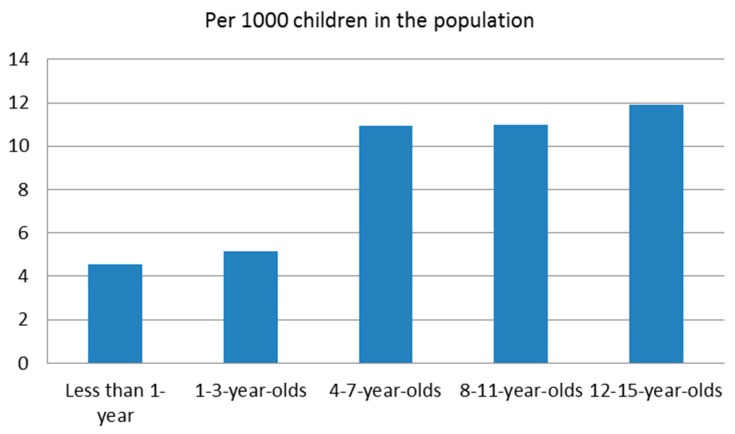
Incidence of a child functioning concern in maltreatment-related investigations.

Descriptive and chi-square analyses revealed many differences between infants and the other four age groups (Table 2). The distribution of child characteristics, including sex and ethnicity are similar across the five different age groups (Table 2). The likelihood of a child welfare worker identifying a child functioning concern increased with age (Table 2 and Figure 2). Infants were significantly less likely to have a child functioning concern identified when compared to children who were early school-aged (4–7), pre-adolescent (8–11), and adolescent (12–15). Infants had the lowest incidence at a rate of 4.53 per 1000. In contrast, adolescents (12–15) had the highest incidence rate at 11.93 per 1000. 

### 3.2. Primary Caregiver Characteristics and Risk Factors

**Figure 3:**
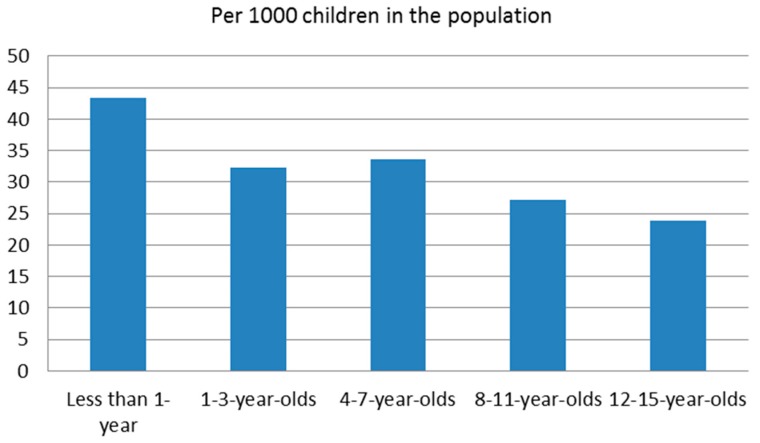
Incidence of a caregiver risk factor in maltreatment-related investigations in Ontario 2013.

Infants had the highest incidence of caregivers identified as having at least one risk factor, with a rate of 43.44 per 1000 (Figure 3). Early school-aged children followed next with an incidence rate of 33.64 per 1000, preschoolers have an incidence rate of 32.33 per 1000, and preadolescents have an incident rate of 27.17 per 1000. Adolescents have the lowest incidence rate of caregivers identified with at least one caregiver risk factor at a rate of 23.78 per 1000.

Chi square tests (Table 2) revealed caregivers of infants were significantly more likely to be younger (21 years of age or under) than caregivers of children in each of the four older age groups. Caregivers of infants were also significantly more likely to be identified as having the following risk factors: drug solvent use, cognitive impairment, mental health issues, having a history of foster care, and having at least one caregiver risk factor identified. The three most common caregiver risk factors identified for caregivers of infants were being a victim of IPV, few social supports, and having a mental health issue (Table 2).

### 3.3. Household Characteristics

Infants were most frequently identified as living in households with at least one hazard, regularly running out of money, and moving two or more times. Chi-square analyses also indicated that infants were significantly more likely to be identified to be living in a household that regularly ran out of money for basic necessities than families of older children. 

### 3.4. Maltreatment, Case Characteristics, and Short-Term Service Outcomes

When compared to all other age groups, infants were most likely to be involved in investigations where there was no specific incident of maltreatment alleged, but there was an allegation of future risk of maltreatment. Infants and pre-school aged children were most frequently investigated for exposure to IPV. Neglect was the most common type of investigated maltreatment, with minimal variation across age groups. Moreover, in comparison to each of the four older age groups, investigations involving infants were significantly more likely to result in court involvement and a transfer to ongoing child welfare services. With the exception of adolescents, investigations involving infants were significantly more likely to result in an out-of-home placement for all other age groups. Infants and adolescents were among the age groups with the highest prevalence of out-of-home placement post-investigation. 

### 3.5. Characteristics Related with Service Referrals for Maltreatment-Related Investigations Involving Infants

Chi-square analyses were conducted in order to determine which child, caregiver, household, and case characteristics influenced the decision to provide a referral for maltreatment-related investigations involving infants. Table 3 shows the characteristics of maltreatment-related investigations involving infants and the eight variables that were significantly associated with the decision to provide referrals for supportive services: the identification of a child functioning issue, younger primary caregiver age, drug/solvent use; few social supports, at least one household hazard, regularly running out of money; and, greater number of moves. The eight variables found to be significant in relation to service referrals were then placed in the binary logistic regression model. 

### 3.6. Predictors of Service Referral for Maltreatment-Related Investigations Involving Infants: Logistic Regression Analysis

There were two primary caregiver characteristics that contributed to the prediction of a service referral for infants in the final model: being a victim of IPV and primary caregiver age. Thus, infants’ exposure to IPV and having a younger caregiver increased the likelihood of a service referral (Table 4). Having a caregiver who was a victim of IPV was the largest contributor to the decision to refer to specialized services. Having a primary caregiver aged 21 years or younger, in comparison to having a primary caregiver 22 years or older, more than doubled the odds of being referred for services (OR = 2.54, *p* < 0.01). The presence of IPV among primary caregivers increased the likelihood of a service referral being made by a factor of 2.81 (OR = 2.81, *p* < 0.01). The omnibus tests of model coefficients *χ*^2^ (8) = 39.24, *p* < 0.001 indicates that the model was significant. The model accounted for approximately 20.0% of the variance on the outcome (Nagelkerke *R*^2^ = 0.20).

### 3.7. Referral to Supportive Services

General areas for possible referrals to supportive services included: parenting and family support; addiction and mental health support; physical health; IPV, legal and victim support; income, food and housing support; cultural services; speech and language; and, recreational services. As Table 5 shows, interesting patterns emerged in the types of referrals provided by child welfare workers by age group. In comparison to all other age groups, families of infants were most commonly referred to a parent support group and in-home family or parenting counseling, and/or addiction counseling. Amongst families of infants, parenting and family support was the most common type of service referred to by child welfare workers.

Families with infants were most likely to receive a referral for drug or alcohol counseling; whereas families with adolescents were most likely to receive referrals for mental health. Overall, families of infants and preschool aged children were most likely to receive referrals for supports for meeting their basic needs, and included income, food, and housing supports. When compared to families of children in older age groups, families with younger children (i.e., infants and preschool aged children) were almost equally likely to be referred for IPV supports. 

## 4. Discussion

Utilizing a Canadian provincial dataset, this study corroborates and extends the existing research. The findings highlight the distinct profile of infants and families investigated by the child welfare system. When comparing maltreatment-related investigations of infants to older children, numerous differences emerged with respect to risks, service needs, the child welfare system’s response, and the types of supportive services referred to by the child welfare system. The findings of this study also provide a broad understanding of the clinical factors that drive the decision to provide a referral to services for infants and their families. The majority of investigations involving infants received a service referral. When compared to older children, maltreatment-related investigations involving infants were significantly more likely to result in a referral for supportive services. These findings are indicative of the child welfare system’s recognition of the complex challenges families of infants are contending with and the importance and necessity of working with services in the community to address them. When comparing caregivers of infants to those of older children, caregivers of infants were significantly more likely to be identified as having at least one functioning issue. Maltreatment-related investigations involving infants were significantly more likely to have an investigating worker identify primary caregiver risk factors that include drug/solvent use, cognitive impairment, and a history of foster care. Alcohol abuse, drug/solvent use, cognitive impairments, mental health issues, few social supports, being a victim of IPV, and having a history of foster care were risk factors that were more common for caregivers of infants. 

When compared to older children, infants were more likely to live in homes that regularly ran out of money for basic necessities, such as food, shelter, and utilities. These findings are consistent with research indicating that families with younger children tend to experience greater socio-economic hardships than older children [36,37]. This finding is concerning given that the literature suggests that low socio-economic status is linked to poor developmental outcomes for younger children as a result of its detrimental impact on the quality of the infant-caregiver relationship [53]. Poverty has also been identified as a risk factor for neglect [29]. This study found that neglect was the primary form of maltreatment alleged in approximately 1 in every 5 maltreatment-related investigations for each age group. The rapidity of brain development during infancy makes infants particularly susceptible to the profound and widespread developmental effects of neglect [29]. 

Infants were also significantly more likely to be investigated for reasons other than specific incidents of alleged maltreatment. Although the type of investigation (risk-only versus maltreatment) was not associated with the decision to provide a referral in this study, previous research suggests that risk-only investigations present with similar household and poverty-related concerns as maltreatment investigations [54]. This raises issues with respect to the operationalization of child welfare’s dual mandate of child safety and well-being for infants, particularly within the context of traditional service delivery models that emphasize protection. A child safety focus tends to prioritize the assessment of risk; whereas, a focus on well-being prioritizes the assessment of child and family needs [50]. Child welfare service decisions, including the decision to refer to services, should be based on a comprehensive assessment that includes consideration of both risk and needs [50]. This study corroborates previous research that suggests that infants and their families are the recipients of the child welfare system’s most intensive responses [37]. When compared to older children, infants were significantly more likely to be transferred to ongoing services and to be the subject of a court application. Both infants had the highest proportion of out-of-home placements, followed by adolescents. Previous research has found that infants are more likely than older children to be placed out-of-home [37]; however, it is important to note that the vast majority of infants in this study (over 90%) remained in the home following a maltreatment-related investigation. Commonly, infants who remain at home following alleged maltreatment experience high rates of re-reporting [55]. Research examining the re-reporting of infants and children following maltreatment-related investigations that considers the Ontario child welfare context is important to informing prevention and intervention efforts [56]. 

Two factors were significant in predicting the decision to refer to services and both were associated with the primary caregiver: being a victim of IPV and younger caregiver age. As in Jud, Fallon, and Trocmé’s study, the presence of IPV had a significant impact on the decision to refer to services [24]. Having a caregiver who was a victim of IPV was the most common caregiver risk factor identified by investigating child welfare workers for all age groups, with the exception of adolescents. In keeping with the findings of Fallon and colleagues, IPV exposure in this study was found to be the most commonly identified concern in maltreatment-related investigations involving infants in Ontario [16]. The finding that service referral decisions were also significantly associated with younger caregiver age is consistent with previous Canadian research [24] and the broader literature indicating that young caregiver age is a risk factor for maltreatment and is associated with the decision to provide ongoing services [16,28,34,57]. It is notable that many variables (e.g., child functioning concern, primary caregiver drug/solvent use, primary caregiver with few social supports) did not contribute to the decision to provide a service referral for infants. It appears that child welfare workers were most clinically concerned about the impact of IPV and younger caregiver age on caregiving skills and ability to meet infants’ needs. Previous research from Ontario conducted by Fallon and colleagues suggests that a differential systems response is required for IPV and this should depend on the type of exposure and the constellation of other risk factors at the child, family, and household levels [58]. Further research is required to better understand the child welfare response to IPV in a Canadian context [58].

The absence of association between caregiver mental health and service referral at the bivariate level is not in keeping with broader Canadian research on infants that has linked caregiver mental health to other service provision decisions, such as the decision to provide ongoing services [16,34], and the decision to place infants out-of-home [48]. Moreover, there is a well-established body of research that suggests that caregiver functioning issues, such as chronic depression, may act to compromise the quality of the infant-caregiver relationship and negatively impact short-term and long-term development [59]. Socio-economic hardship risk variables (e.g., running out of money for basic necessities, primary income, unsafe housing/household hazards) did not seemingly impact the likelihood of service referrals in this model. It may be that the measures informing socio-economic disadvantage are somewhat crude and socio-economic status is generally quite low for many families that come to the attention of the child welfare system in Ontario. 

Disparities in the decision to provide and utilize services have been linked to child race and ethnicity [5,10,60]. In this study, child ethnicity was not significant at the bivariate level. This is in keeping with Jud, Fallon, and Trocmé’s study that found that ethnicity did not influence the decision to provide ongoing services or a referral for supportive services [24]. The role of child race in service referral decision-making is not well understood [5] Further research is needed to explore racial and ethnic disparities in service provision decisions, including the decision to refer to services regarding infants within a Canadian child welfare context. 

In keeping with previous research [16], this study revealed that when compared to older children, infants in Ontario are less likely to be identified as having a child functioning issue. Although found to be significantly associated with service referrals at the bivariate level for infants, the identification of a child functioning concern did not predict a service referral in the multivariate model. The literature compellingly suggests that the mental health needs of infants and young children involved with the child welfare system are under-identified and untreated. Referral to appropriate services for families and children starts with the accurate identification of needs. There is no comprehensive or systematic developmental screening strategy in place in Ontario for infants involved with the child welfare system. Standardized, reliable, and valid measures may assist child welfare workers in the assessment and identification of need of early intervention services. There were age-specific differences in the types of services families were referred to by investigating child welfare workers. This study revealed that families of infants and young children (0–3 years old) were most commonly referred to services that relate to IPV and parenting/family support, such as support groups and/or counselling. Infants and younger children (ages 0–3 years) were also more commonly referred to income, food, and housing support services (e.g., social assistance, food bank, and shelter services) than older children. The importance of adopting a differential approach to child welfare policy, practice, and research that considers child age has been forwarded and is supported by the findings of this study [61]. Ontario has not yet implemented wide-scale programs with respect to differential response options [16,45]. Together with other studies, these findings lead to questioning whether an alternative or differential response approach with infants should be a consideration given their distinct clinical profile, risks, and need for supportive services.

In principle, service referrals should be primarily driven by case or clinical factors; however, child welfare service decisions are complex and can be influenced by factors at other levels, including worker, organizational, and the broader environment [62]. In addition, organization-level factors and regional variations have been found to influence the likelihood of service referrals [24]. The availability and accessibility of services may also be influencing child welfare workers’ decisions to refer to services to and the types of services are referred to. As such, both availability and accessibility may be influenced by geographic location, which may in turn influence the ability of families to utilize community services [63]. Location may be a proxy measure for several underlying organizational constructs, including differential access to resources and partnerships between social service agencies and child welfare organizations [64]. Differences in child welfare services and geographic location or jurisdiction have not been adequately addressed in the literature [64]. Thus, influences at the organizational and structural levels require further research, particularly with respect to maltreatment-related investigations involving infants [24].

In Ontario, infant and childhood mental health has been identified as, “… an issue that requires further policy development to ensure the availability and accessibility of optimal and consistent services across the province” (p. 5) [65]. To date, there is no provincial strategy that has focused on the mental health needs of infants and young children in Canada [65]. A key recommendation made for immediate policy development includes the provision of targeted supports to populations at-risk and working with an inter-generational intervention model [65]. Clinton and colleagues noted that infant-caregiver dyad is not the primary client focus in Ontario services, as infants and caregivers are treated as distinct entities [65]. 

### Study Limitations

There are several limitations to this study that must be considered when interpreting the results. Data collected from the OIS is collected directly from the investigating child welfare worker and are not independently verified. The data is representative of an investigative period of thirty days after the case has opened. The study is cross-sectional and does not track longer-term service events that occurred beyond the initial investigation. The three-month sampling period is considered optimum to ensuring high participation rates and compliance with study procedures [51]. Consultations with service providers have indicated that case activity during the three-month period is reflective of the year; however, follow-up studies are needed to explore the extent of the possible impact of seasonal variation in types of cases reported on estimates [51]. The sample size of maltreatment-related investigations involving infants may have precluded some significant relationships from emerging. Types of services referred to were categorized broadly by family, not by child or caregiver, and provide a broad understanding of risk and needs. The OIS does not track specific referral actions or strategies used by child welfare workers (e.g., providing families with names and numbers, assisted caregiver with the referral). The OIS does not track whether services were received as a result of referral made by the worker during the investigative period. The OIS tracks whether a referral has been made and they type of services referred to. It is unknown whether families were engaged in other community services during this period. Moreover, the amount of variance explained by this model was small, with a large proportion of variance unaccounted for.

## 5. Conclusions

This study contributes to the minimal knowledge base relating to infants served by the child welfare system within a Canadian context. Despite the study limitations in tracking the progress of infants through the child welfare system, this study marks an important step to moving towards a better understanding of what investigations will effectively address the needs of vulnerable infants and their families. To date, the OIS is the only source of aggregated data in the province of Ontario and the findings are a reminder of the importance of provincial data in exploring and advocating for possible gaps in supports and resources for child-welfare involved children. Child welfare policy is set by the provincial government that also sets policies and funds for other allied sectors (e.g., children’s mental health) that are integral to effective service provision. Greater understanding of the factors associated with service referrals and the types of services referred to can assist in aligning, organizing, and targeting child welfare and community services to address the risks and needs of child-welfare involved children and families. 

A referral for services can be viewed as a critical step to enhancing infants’ safety and well-being, and should be predicated upon the accurate identification of infant and family risks and needs. There are concerns that the child welfare system missing opportunities to identify, and thereby, ameliorate infants’ well-being given the extant literature and this study’s findings of low identification of infant functioning. Further research within a Canadian context is necessary in order to address these concerns. Moreover, adequately addressing infant well-being requires services that are accessible, available, and effective. As the findings suggest, infants are a distinctly vulnerable group of children involved with the child welfare system and require urgent and coherent attention at policy and practice levels within the field of child welfare and across multiple sectors. 

## Figures and Tables

**Table 1:** Variable definitions and codes.

Variable	Description	Measurement
**Outcome**		
Referral to supportive services	Workers were asked to indicate if a referral was made to any services internal to the child welfare system or externally to community services (e.g., parent support group) for any family member.	Dichotomous variable:
		1 Referral for services
		0 No referral for services
**Predictors**		
Child Characteristics		
Child sex	Worker identified the sex of the investigated child.	Dichotomous variable:
		1 Male
		0 Female
Child ethno-racial group	Workers were asked to indicate the ethno-racial background of the child (Black, Latin American, Arab, Aboriginal, Asian). Ethno-racial categories developed by Statistics Canada.	Dichotomous variable:
		1 Ethnic minority
		0 White
Child functioning	Workers were asked to note up to eighteen child functioning concerns. Six of eighteen dichotomous child functioning variables are relevant to infants: failure to meet developmental milestones, attachment issues, intellectual/developmental disability, FAS/FAE, positive toxicology at birth, physical disability. This analysis noted whether the worker examined at least one of these six concerns.	1 At least one child functioning concern noted.
		0 No child functioning concerns noted
Caregiver Characteristics		
Primary caregiver age	Workers were asked to indicate the age category of the primary caregiver.	Categorical variable:
		1 21 years and under
		2 22 years and up
Primary caregiver risk factors	Workers could note up to nine functioning concerns for the primary caregiver. Concerns were: alcohol abuse, drug/solvent abuse, cognitive impairment, mental health issues, physical health issues, few social supports, victim of domestic violence, perpetrator of domestic violence, and history of foster care/group home.	Nine dichotomous variables:
		1 Suspected or confirmed
		0 No or unknown
Primary income of caregiver	Workers were asked to indicate the primary source of the primary caregiver’s income.	Categorical variable:
		1 Full time/Part time
		0 Other benefits/unemployment/
		No income
Household characteristics		
No second caregiver in the home	Workers were asked to describe up to two caregivers in the home. If there was only one caregiver described there was no second caregiver in the home.	Dichotomous variable:
		1 No second caregiver in home
		0 Second caregiver in home
Household hazards	Workers were asked to note if the following hazards were present in the home at the time of the investigation: accessible weapons, accessible drugs, production/trafficking of drugs, chemicals/solvents, used in drug production, other home injury hazards, and other home health hazards.	Dichotomous variable:
		1 At least one household hazard
		0 No household hazard
Household regularly runs out of money	Workers were asked to note if the household regularly runs out of money for food, housing and/or utilities in the last six months.	Dichotomous variable:
		1 Noted
		0 Not noted
Number of moves	Workers were asked to note the number of moves the household had in the past six months.	2 2 or more moves
		1 One or more moves
		0 None
Case characteristics		
Previous openings	Worker indicated if there were one or more previous child protection openings.	1 One or more previous openings.
		0 No openings
Type of investigation	Workers were asked to indicate if the investigation was conducted for a specific maltreatment incident (maltreatment investigation), or if it was to assess a risk of maltreatment only (risk-only investigation).	1 Maltreatment investigation 2 Risk-only investigation

**Table 2:** Prevalence and bivariate analyses of investigation characteristics by child age for maltreatment-related investigations in Ontario in 2013.

Variable	Child Age					
	Less than 1Year- Old	1–3- Year- Olds	4–7- Year- Olds	8–11- Year- Olds	12–15- Year- Olds	*χ*^2^
	*n* = 7915	*n* = 21,801	*n* = 36,730	*n* = 29,907	*n* = 28,928	
**Child characteristics**						
Child Sex (female)	49.30%	49.40%	48.50%	45.10%	51.30%	NS
Child Ethnicity						
Ethnic minority	30.90%	36.10%	35.60%	35.30%	34.90%	NS
White	69.10%	63.90%	64.40%	64.70%	65.10%	
**Child Functioning Concerns**						
At Least One Child Functioning Concern	7.80%	10.10%	17.10%	21.30%	26.20%	*p* < 0.001 b;c;d
**Primary caregiver characteristics**						
Primary Caregiver Age						
21 years or under	31.30%	9.80%	0.70%	0%	0.10%	*p* < *0*.001 a;b;c;d
22 years or more	68.70%	90.20%	99.30%	100%	99.90%	
Primary Caregiver Risk Factors						
Alcohol Abuse	10.60%	6.50%	6.80%	7.30%	5.70%	*p* < 0.01 d
Drug/Solvent Use	18.80%	8.60%	6.20%	6.40%	4.10%	*p* < 0.001 a;b;c;d
Cognitive Impairment	8.80%	3.90%	3.80%	4.30%	2.60%	*p* < 0.01a;c
						*p* < 0.001 b;d
Mental Health Issues	31.10%	22.10%	19.00%	19.50%	20.60%	*p* < 0.01 a;
						*p* < 0.001 b;c;d
Physical Health Issues	5.90%	4.70%	5.60%	6.80%	9.00%	NS
Few Social Supports	32.80%	30.90%	22.30%	21.50%	24.50%	*p* < 0.001 b;c
						*p* < 0.01 d
Victim of IPV	37.60%	33.50%	25.60%	24.80%	20.00%	*p* < 0.001 b;c;d
Perpetrator of IPV	10.80%	11.80%	7.30%	7.80%	6.10%	*p* < 0.01 d
History of Foster Care	13.00%	7.40%	4.10%	4.00%	2.20%	*p* < 0.01 a
						*p* < 0.001 b;c;d
At least one caregiver functioning concern	74.30%	63.10%	52.30%	52.80%	52.30%	*p* < 0.001 a;b;c:d
**Household characteristics**						
No Second Caregiver in Home	30.40%	31.20%	37.40%	32.60%	38.10%	*p* < 0.01d
Primary Income						
Full-time/part-time/seasonal	12.80%	35.70%	49.60%	57.20%	64.00%	*p* < 0.001 a;b;c;d
Other Benefits/Unemployment/No income	87.20%	64.30%	50.40%	42.80%	36.00%	
At Least One Household Hazard	8.80%	4.80%	4.10%	5.10%	3.40%	*p* < 0.01 a;b
						*p* < 0.001 d
Household Regularly Runs Out of Money	15.70%	9.20%	7.40%	7.90%	7.30%	*p* < 0.01 a
						*p* < 0.001 b;c;d
Number of Moves						
No Moves	50.00%	62.20%	69.80%	77.00%	79.10%	*p* < 0.001 a
						*p* < 0.001 b;c;d
One Move	32.50%	29.80%	25.00%	19.00%	17.10%	
Two or More Moves	17.40%	8.00%	5.20%	4.00%	3.80%	
**Maltreatment characteristics**						
Type of Maltreatment-Related Investigation *Maltreatment*						*p* < 0.001 a;b;c;d
Physical Abuse	2.10%	12.40%	24.00%	24.30%	21.00%	
Sexual Abuse	1.20%	1.10%	3.80%	3.60%	5.02%	
Neglect	20.40%	20.50%	21.60%	21.60%	21.70%	
Emotional Maltreatment	5.40%	7.40%	8.30%	8.20%	10.60%	
Exposure to (IPV)	31.20%	31.30%	24.40%	23.90%	20.40%	
*Risk*	39.80%	27.30%	18.00%	18.30%	21.20%	
**Case characteristics and service outcomes**						
At least one previous case opening (family level)	43.60%	60.50%	63.20%	68.20%	72.70%	*p* < 0.001 a;b;c;d
Child previously investigated for alleged maltreatment (child level)	16.90%	45.90%	56.90%	64.20%	67.70%	*p* < 0.001 a;b;c;d
Opened for ongoing services	39.80%	25.70%	21.60%	23.60%	26.30%	*p* < 0.001 a;b;c;d
Child welfare court	8.80%	1.90%	1.90%	1.50%	3.30%	*p* < 0.001 a;b;c;d
Placement	8.60%	1.20%	2.20%	2.80%	5.90%	*p* < 0.001 a;b;c
Investigations with a service referral	57.2%	46.0%	39.0%	45.4%	46.3%	*p* < 0.001 a;b;c;d

Source: 2013 Ontario Incidence Study of Reported Child Abuse and Neglect; a = statistically significant difference in service referrals between infants (less than 1 year old) and 1–3-year-olds. b = statistically significant difference in service referrals between infants and 4–7-year-olds. c = statistically significant difference in service referrals between infants and 8–11-year-olds. d = statistically significant difference in service referrals between infants and 12–15-year-olds (*p* < 0.01; *p* < 0.001), NS = not statistically significant. Chi-square analyses were conducted with the normalized sample weight. Estimated number of provincial investigations, *n* = 125,281.

**Table 3:** Referral-related characteristics in maltreatment-related investigations involving infants referred for services in Ontario in 2013.

Variable	Referral for Services		
	Estimate	%	*χ* ^2^
**Child Characteristics**			
Child sex			
Male	2302	57.6%	0.01
Female	2228	57.3%	
Child ethnicity			
Ethnic minority	1212	50.6%	3.65
White	3310	61.8%	
Child functioning issue			
At least one identified	472	76.9%	4.41 *
**Caregiver Characteristics**			
Primary caregiver age			
21 years and under	1638	66.4%	5.29 *
22 years and up	2855	52.8%	
Primary caregiver risk factors			
Alcohol Abuse	556	66.0%	1.15
Drug/Solvent Use	1087	73.0%	7.79 **
Cognitive Impairment	488	69.8%	2.16
Mental Health Issues	1590	64.7%	3.23
Physical Health Issues	321	68.9%	1.03
Few Social Supports	1743	67.1%	6.29 *
Victim of IPV	2124	71.3%	15.99 ***
Perpetrator of IPV	517	60.6%	0.23
History of Foster Care	580	56.3%	0.04
Primary income of caregiver			
Full time/Part time	457	48.3%	3.12
Other benefits/unemployment/no income	4016	62.2%	
**Household characteristics**			
No second caregiver in the home	1470	61.1%	0.96
Household hazards	566	85.1%	9.18 ***
Household regularly runs out of money	749	72.7%	3.86 *
Number of moves			
None	1499	46.0%	24.45 ***
One move	1604	75.6%	
Two or more moves	849	74.6%	
**Case characteristics**			
Previous openings	2096	60.8%	1.26
Type of investigation			
Maltreatment	2598	54.5%	1.53
Risk	1932	61.3%	

Source: 2013 Ontario Incidence Study of Reported Child Abuse and Neglect; * *p* < 0.05; ** *p* < 0.01; *** *p* < 0.001.

**Table 4:** Logistic regression model predicting service referrals for maltreatment-related investigations involving infants in Ontario in 2013.

Variable	B (SE)	Wald	OR	95% CI
**Child characteristics**				
At least one child functioning issue	0.39 (0.53)	0.53	1.47	0.52–4.16
**Primary caregiver characteristics**				
Primary Caregiver Age (22 years or more)				
21 years or under	0.93 (0.32)	8.52	2.54 **	1.36–4.75
Primary Caregiver Risk Factors				
Drug/Solvent Abuse	0.32 (0.40)	0.65	1.38	0.63–3.02
Few Social Supports	0.57 (0.32)	3.16	1.76	0.94–3.28
Victim of IPV	1.04 (0.31)	11.32	2.83 **	1.54–5.18
**Household characteristics**				
At Least One Household Hazard	0.73 (0.59)	1.54	2.07	0.66–6.51
Household Regularly Runs Out of Money	0.12 (0.41)	0.08	1.12	0.52–2.42
Number of Moves (No Moves)				
One Move	0.45 (0.32)	2.04	1.60	0.85–2.91
Two or More Moves	0.06 (0.50)	0.16	1.06	0.42–2.67
Omnibus Chi-Square Test	39.24			
*p*-value	*p* < 0.001			
Nagelkerke R-square	0.20			
% Classified correctly	67.6			
−2 Log Likelihood	301.54			

Source: 2013 Ontario Incidence Study of Reported Child Abuse and Neglect; SE, Standard error; OR, Odds Ratio; CI, Confidence Interval; * *p* < 0.05; ** *p* < 0.01; *** *p* < 0.001.

**Table 5:** Type of service referrals in maltreatment-related investigations by age group in Ontario in 2013.

Variable	Child Age				
Type of Service(s) Referred to	Less than 1- Year Old	1–3- Year- Olds	4–7- Year- Olds	8–11- Year- Olds	12–15- Year- Olds
	*n* = 4530	*n* = 9755	*n* = 14,104	*n* = 13,400	*n* = 13,148
**Parenting or family support**					
Parent support group	22.7%	14.2%	16.1%	10.9%	14.9%
In-home family/parent counseling	20.8%	12.8%	13.5%	11.9%	11.9%
Other family/parent counseling	22.6%	35.8%	43.4%	44.8%	53.8%
Child or day care	6.3%	16.0%	5.8%	1.7%	0.8%
**Addiction and mental health support**					
Drug or alcohol counseling	17.4%	14.3%	11.3%	11.9%	11.2%
Psychiatric or psychological services	10.4%	14.5%	15.4%	16.0%	19.5%
Physical health support (medical/dental)	6.4%	6.7%	4.4%	5.3%	5.0%
**IPV, legal and victim support**					
IPV services	24.0%	24.7%	19.6%	18.1%	14.0%
Victim support program	4.7%	11.1%	9.4%	7.3%	6.4%
Legal	13.3%	16.5%	9.8%	10.2%	7.1%
**Income, food and housing support**					
Welfare or social assistance	7.8%	6.0%	3.1%	1.4%	3.3%
Food bank	7.8%	7.0%	6.4%	5.7%	6.0%
Shelter services	5.5%	11.4%	7.0%	4.2%	5.7%
Housing	9.6%	12.8%	4.8%	6.5%	4.0%
Cultural services	2.7%	2.8%	3.4%	4.1%	5.7%
Speech or language	0.8%	5.6%	1.9%	1.2%	1.1%
Recreational services	3.7%	6.7%	5.3%	5.9%	4.2%

Source: Ontario Incidence Study of Reported Child Abuse and Neglect 2013; Percentages do not add up to 100% because investigating child welfare workers could identify more than one service referred to.
